# Humoral and cellular immunogenicity and efficacy of a coxsackievirus A10 vaccine in mice

**DOI:** 10.1080/22221751.2022.2147022

**Published:** 2023-01-17

**Authors:** Huan-Huan An, Meng Li, Rui-Lun Liu, Jie Wu, Sheng-Li Meng, Jing Guo, Ze-Jun Wang, Sha-Sha Qian, Shuo Shen

**Affiliations:** a Wuhan Institute of Biological Products Co. Ltd., Wuhan, People’s Republic of China; b College of Medical Laboratory Science, Guilin Medical University, Guilin, People’s Republic of China

**Keywords:** CV-A10 vaccine, humoral and cellular immunogenicity, T cell epitope, mouse model, active immunization-challenge, efficacy

## Abstract

Coxsackievirus A10 (CV-A10) has become one of the major pathogens of hand, foot and mouth disease (HFMD), and studies on the vaccine and animal model of CV-A10 are still far from complete. Our study used a mouse-adapted CV-A10 strain, which was lethal for 14-day-old mice, to develop an infected mouse model. Then this model was employed to establish an actively immunized-challenged mouse model to evaluate the efficacy of a formaldehyde-inactivated CV-A10 vaccine, which was prepared from a Vero cell-adapted strain. CV-A10 vaccine at a dose of 0.5 or 2.0 μg was inoculated intraperitoneally in neonatal Kunming mice on the third and ninth day. Then the mice were challenged on day 14. The survival rate of mice immunized with 0.5 or 2.0 μg vaccine were 90% and 100%, respectively, while all Alum-inoculated mice died. Compared to those in the two vaccinated groups, the Alum-inoculated mice showed severe pathological damage, strong viral protein expression and high viral loads. The antisera from vaccinated mice showed high level of neutralizing antibodies against CV-A10. Meanwhile, three potential T cell epitopes located at the carboxyl-terminal regions of the VP1 and VP3 were identified and exhibited CV-A10 serotype-specific. The humoral and cellular immunogenicity analysis showed that immunization with two doses of the vaccine elicited CV-A10 specific neutralizing antibody and T cell response in BALB/c mice. Collectively, these findings indicated that this actively immunized-challenged mouse model will be invaluable in future studies on CV-A10 pathogenesis and evaluation of vaccine candidates.

## Introduction

During the past two decades, hand, foot and mouth disease (HFMD) has been prevailing in the Asia-Pacific regions and the disease frequently occurs in children under five years of age [1,2]. Many members of the species *Enterovirus A* are the main causative pathogens of HFMD, including enterovirus A71 (EV-A71), coxsackievirus A16 (CV-A16), CV-A6 and CV-A10 [3]. Previous epidemiological studies revealed that EV-A71 and CV-A16 primarily accounted for global HFMD outbreaks. However, incidences of CV-A10 infections have gradually increased, in countries such as Finland, Singapore and mainland China [4–6]. CV-A10 infections accounted for 11.56% of HFMD cases in Xiangyang, China from October 2016 to December 2017 and 25% in Guangzhou, China in 2018 [7,8]. Normally, infection of CV-A10 causes only mild and self-limiting disease, but a small proportion of patients experience severe life-threatening complications like meningitis, encephalitis and death [9,10]. In addition, CV-A10 has a potential for co-infection and recombination with other co-circulating enteroviruses such as EV-A71, CV-A16 and CV-A6 [4,7,8,11]. These findings indicate that CV-A10 has emerged as one of the prominent causative agents of HFMD, which poses a challenge to the prevention and control of the disease.

Although formaldehyde-inactivated EV-A71 vaccines have been approved for marketing in China [12], these vaccines do not confer a cross-protective effect against other enterovirus serotypes [7]. Hence, it is imperative to develop a multivalent HFMD vaccine including major prevalent pathogenic enteroviruses in the future. As for CV-A10, there is a lack of specific drugs or vaccines in the market, so it is vitally important to investigate infection mechanism and develop therapeutic drugs and prophylactic vaccines for CV-A10 infection. Currently, researchers have attempted to develop CV-A10 vaccines by employing different approaches, such as traditionally inactivated whole-virus vaccines (formaldehyde-inactivated or β-propiolactone-inactivated) [13–17], virus-like particles (VLPs) [18,19] and subunit vaccines [20]. For the CV-A10 inactivated vaccines, the clinical isolate CVA10-TZ3-P5 [15] was MRC-5 cell adapted, CVA10-25 [16] and CVA10/S0148b [17] were Vero cell adapted. Both cells were allowed for human vaccine production. The other evaluated clinical isolate TA151R [13] and CVA10-FJ-01 [14] strains were RD cell-adapted strains.

Meanwhile, various animal models have been established for CV-A10 to investigate the pathogenesis and evaluate the efficacy of vaccines or antivirals, including mice [13,14,16–18,21], gerbils [15] and non-human primates [22]. The vaccine protectiveness is often measured by passive transferring sera from immunized adult mice to newborn suckling mice and examining the effectiveness on subsequent viral challenge. Only Shen [17], Zhang [21] and Chen [15] groups have reported actively immunized-challenged models, based on a lethal CV-A10 challenge in a 12-day-old ICR mouse, 10-day-old ICR mouse and 14-day-old gerbil, respectively. Evaluation of vaccine candidates that mainly focuses on humoral and cellular immunogenicity is less investigated.

In this study, an inactivated Vero cell-adapted CV-A10 vaccine candidate was developed, and both humoral and cellular immunogenicity were investigated. A 14-day-old Kunming mice infection model was established and employed to evaluate the efficacy of the CV-A10 vaccine, using a mouse-adapted challenge strain. The results of our studies have revealed that CV-A10 vaccine induced the production of antibodies and cytokines, and actively immunized antibodies completely protected against viral challenge in 14-day-old Kunming mice.

## Materials and methods

### Ethics statement

This study was performed in compliance with the requirements of the Animal Ethics Procedures and Guidelines of the People’s Republic of China [23]. All specific pathogen-free Kunming or BALB/c mice and facilities were under the control of the Wuhan Institute of Biological Products (WIBP). The animal procedures were approved by the Animal Ethics Committee of the WIBP (WIBP-AII 382021005 and 382021006).

### Cells and viruses

Human rhabdomyosarcoma (RD) cells and African green monkey kidney (Vero) cells were grown as described previously [24]. CV-A10 JZ-001 was isolated from a child who contracted HFMD in Jingzhou, China in 2014. CV-A10-L7 was isolated from specimens of HFMD patients in Xiangyang, China in 2017. Cell culture harvests of CV-A10 were subjected to three freeze–thaw cycles, clarified by centrifugation at 3900 × g for 10 min at 4°C and stored at −80°C. All viruses were titrated for the 50% cell culture infective dose (CCID_50_) assay using the Reed and Muench method [25].

### Preparation of inactivated CV-A10 vaccine

For the CV-A10 vaccine preparation, parental CV-A10 JZ-001 was first isolated and passaged in RD cells for 6 generations, followed by adaptation to Vero cells and then passaged 6 times. The viral stock of the 6th passage in Vero cells was purified by three consecutive plaque-to-plaque purification to obtain the CV-A10-L12 clone. The full genome of CV-A10-L12 was sequenced and an infectious cDNA clone was constructed into pBR322 vector via *de novo* synthesis by Genscript Biotech Corporation (Nanjing, China). Then the vector was linearized and used to perform the *in vitro* T7 RNA polymerase transcription experiment, using the RiboMAX^TM^ large-scale RNA production system (Promega, USA). Following purification, the RNA transcript was transfected with the transfection reagent Lipofectamine 2000 (Thermo Fisher Scientific, USA) in Vero cells. The rescued CV-A10-L12 virus was harvested and propagated in Vero cells for improving virus titres. Then CV-A10-L12 was cultured in Vero cells in a 10-layer cell factory and subjected to freeze–thaw three times. The purification of CV-A10-L12 particles was performed as previously described [26]. The fractions of empty and full particles (EP and FP) at CsCl density gradient (1.30 g/ml) centrifugation were collected, and the concentrations of EP or FP and their natural ratio were determined by Pierce bicinchoninic acid protein assay (Thermo Fisher Scientific, USA). After formaldehyde inactivation, EP and FP were mixed in the natural ratio and the mixture was adsorbed to 10 mg/ml alum adjuvant (Croda Denmark Alhydrogel^®^) and PBS to produce an emulsion. The final vaccine (0.5 µg/dose or 2.0 µg/dose) formulation was shaken for 2 h and stored at 4°C until use.

### Characterization of inactivated CV-A10 particles

SDS-PAGE and Western blotting analysis of the purified inactivated CV-A10 particles (EP, FP and their mixture component named MP) were performed as described previously [26]. The membranes were probed by anti-CV-A10 whole virus, VP1, VP2, VP3 antibodies (developed in our laboratory) and anti-β-actin antibodies (purchased from Abcam). The morphology of CV-A10 particles was analysed by negative staining electron microscopy. Briefly, purified viruses were adsorbed to 200 mesh carbon-coated copper grids and inoculated for 5 min at room temperature. The grids were washed with PBS, stained for 5 min with 1% phosphotungstic acid (pH 7.0) and air dried overnight. The particles on grids were examined under a transmission electron microscopy.

### Acquirement of CV-A10 challenge virus

Mouse adaptation experiments were performed using a RD cell isolate (CV-A10-L7) as described previously for generation of mouse-adapted CV-A2 and CV-A5 [26,27]. A mouse-adapted strain, CV-A10-M14, was obtained via the intracranial (i.c.) route in our lab. The viral stock was cultured in RD cells in a 10-layer cell factory and concentrated by ultrafiltration for determination of the lethal dose for 14-day-old Kunming mice through intraperitoneal (i.p.) route.

### Mouse infection experiments

To evaluate the susceptibility of mice of different ages to CV-A10, 12- and 14-day-old Kunming mice (*n* = 10 in each group) were challenged intraperitoneally (i.p.) with CV-A10-M14 strain (9.5 × 10^8^ CCID_50_ per mouse in a 300 µl volume). To evaluate the median lethal dose (LD_50_) of the virus in infected suckling mice, CV-A10-M14 was diluted in a 10-fold series to 9.5 × 10^8^–9.5 × 10^5^ CCID_50_/mouse/300 µl and 14-day-old Kunming mice were injected through i.p. route. The control mice were inoculated with an equal volume of culture medium. The body weights, clinical scores and survival rates of control or infected mice were monitored daily for 14 dpi (days post-infection). According to previous clinical scores [28], the grades were as follows: 0, healthy; 1, ruffled hair and hunchbacked; 2, limb weakness; 3, single limb paralysis; 4, double limbs paralysis; 5, death. The LD_50_ was calculated as described by the Reed and Muench method [25].

### Immunogenicity of inactivated CV-A10 vaccine in mice

Groups of 8 female BALB/c mice (6∼8 weeks old) were i.p. injected on weeks 0 and 2 at doses of 0.5 µg or 2.0 µg of inactivated CV-A10 vaccine in a volume of 500 µl. The control group was inoculated with an identical volume and concentration of Alum, which was diluted by PBS. The immunized mice were bled for antibody measurement on weeks 2, 4, 6, 8, 10 and 18. The mouse sera were inactivated at 56°C for 30 min and stored at −80°C until analysis. On week 18, the mice were euthanized following the last bleeding.

### Active immunization and virus challenge

Kunming mice at the age of three days were randomly divided into three groups (*n* = 10 per group). The first and second groups of mice were immunized i.p. with 0.5 µg or 2.0 µg inactivated CV-A10 vaccines in 100 µl, respectively. The third negative control group received an identical volume and concentration of Alum, which was mixed with PBS. Six days later (9-day-old), the mice were boosted with the same dose of antigens. All mice were challenged i.p. with CV-A10-M14 at a dose of 4.8 × 10^9^ CCID_50_/mouse when they were 14-day-old. After challenge, the mice were monitored daily for survival rate, body weight and clinical scores for 14 days. The blood and tissue samples of the five mice euthanized from each group were collected on 0, 4 and 14 days post-infection (dpi). Sera were inactivated at 56°C for 30 min before use. Organs including heart, liver, spleen, lung, kidney, intestine, brain and hindlimb muscle were taken and divided into two parts. One part was weighed and stored at −20°C for virus load assay via quantitative real-time PCR (qRT-PCR). The other part was fixed with 4% paraformaldehyde and used for histopathological examination and immunohistochemistry assay.

### Neutralizing antibody (NtAb) assay

Measurement of neutralizing antibody was performed as previously described [24]. Briefly, serum samples from mice were two-fold serially diluted in two duplicates and mixed with equal volume of CV-A10-L12 (50 µl, 2,000 CCID_50_/ml) in 96-well plates at 37°C for 2 h. Virus-serum mixtures were added to the RD cells and incubated at 37°C for 7 days. The neutralizing antibody titre was calculated as the reciprocal of the highest serum dilution that exhibited a complete inhibition of the cytopathic effect (CPE) in 50% of wells.

### Quantitative real-time PCR

Samples were collected from the Kunming mice in the active immunization and challenge experiment. The organs and tissues in 1 g/10 ml MEM medium were homogenized. After centrifugation, viral RNA (vRNA) was extracted from serum and tissue supernatants with QIAamp viral RNA mini kit (Qiagen, Germany) by following the manufacturer’s recommended protocol. The virus loads were determined by qRT-PCR using One Step PrimeScript III RT-qPCR Mix kit (Takara, Japan). The conditions of the reaction were described previously [24]. The plasmid containing a 121 bp fragment of CV-A10-M14 VP1 was constructed and used as a template for T7 *in vitro* transcription to produce the RNA standard. A standard curve generated from serially diluted RNA standard was used to quantify the copy numbers of CV-A10-M14 in tested organs and tissues. The primers used in this study were CV-A10 VP1 F (5′-GGTGTTGGAAACTACTATC-3′), CV-A10 VP1 R (5′-ACCCATAATGTCTATGTCC-3′) and CV-A10 VP1 Probe (5′-FAM-ACCACTTCTTCTCCCGCTCTG-BHQ_2_-3′).

### Histopathologic and immunohistochemistry (IHC) analysis

Various tissues and organs were fixed in 4% paraformaldehyde. The histopathological examination (H&E) and immunohistochemical (IHC) staining were analysed by the Biofavor biotech corporation (China). The healthy Kunming mice at the age of 28 days were used as the negative controls. The experiments were performed according to the company’s protocol, which was described in the previous study [24], except that a polyclonal rabbit anti-CV-A10 whole virus antibody was used as the detection antibody in IHC assay.

### Synthetic peptides

To evaluate T cell responses against CV-A10, the four structural proteins from CV-A10-L12 were selected as a template sequence for overlapping peptide design. A total of 106 15- to 19-mer peptides which overlapped by 10 aa at both ends and spanning the VP1-VP4 structural proteins were synthesized by GenScript biotech corporation (Nanjing, China) (Supplemental Table S1). All the peptides were >90% pure as assessed by high-performance liquid chromatography (HPLC). The peptides were firstly dissolved in ultrapure water or dimethyl sulfoxide (DMSO, Sigma, USA) to a concentration of 1 mM and stored at −80°C. Before use, the stock solution was diluted in Serum-Free Medium for ELISPOT (MabTech, Sweden) to obtain a 10 μM as working concentration.

### Cytokine assays

Two groups (*n* = 5 per group, 6∼8 weeks old) of BALB/c mice were i.p. immunized twice with the inactivated CV-A10 vaccine at a dose of 0.5 µg or 2.0 µg at a two-weeks interval. The control group was i.p. injected with Alum alone. Fourteen days after boosting immunization, three mice from each group were sacrificed to collect spleens for ELISPOT assay. Single-cell spleen suspension was obtained using Mouse 1 × Lymphocyte Separation Medium (DAKEWE, China), and then washed with RPMI 1640 medium (Gibco, Germany). Washed cells were diluted to 2 × 10^6^ cells per ml in Serum-Free Medium for ELISPOT and added to the wells of 96-well Mouse ELISpotPLUS plates (Mabtech) in 100 μl volume (2 × 10^5^ cells per well). Single or mixed CV-A10 peptides which contained 5 adjacent peptides were added to each well at a final concentration of 10 μM for individual peptides. Negative and positive controls were included by incubating lymphocyte without peptide (background control) and with phytohemagglutinin (PHA), respectively. Plates were incubated at 37°C and 5% CO_2_ for 36 h. The number of IL-2, IL-6, TNF-α and IFN-γ secreting cells (SFC, “spot forming cells”) was scanned and counted with ImmunoSpot 5.1 software by CTL S6 Universal-V Analyzers (Cellular Technology Limited, USA). For each individual peptide, the assay was run in triplicate and a response to a peptide was considered positive if the mean of detected SFC was ≥ 50 per 2 × 10^5^ input cells after subtracting the mean ± standard deviation of the background count.

### Statistical analysis

GraphPad Prism 8 software (GraphPad Software Inc., USA) was employed to statistically analyse the survival rate, body weight, clinical score, cytokine expression, the neutralizing antibody titre, as well as the viral load. One-way analysis of variance (ANOVA) with Dunnett’s multiple comparisons test were used. Statistical significance was indicated as follows: n.s., not significant (*P* ≥ 0.05); *, 0.01 ≤ *P* < 0.05; **, *P *< 0.01; ***, *P* < 0.001 and ****, *P* < 0.0001.

## Results

### Purification and characterization of inactivated CV-A10 vaccine

**Figure 1.**
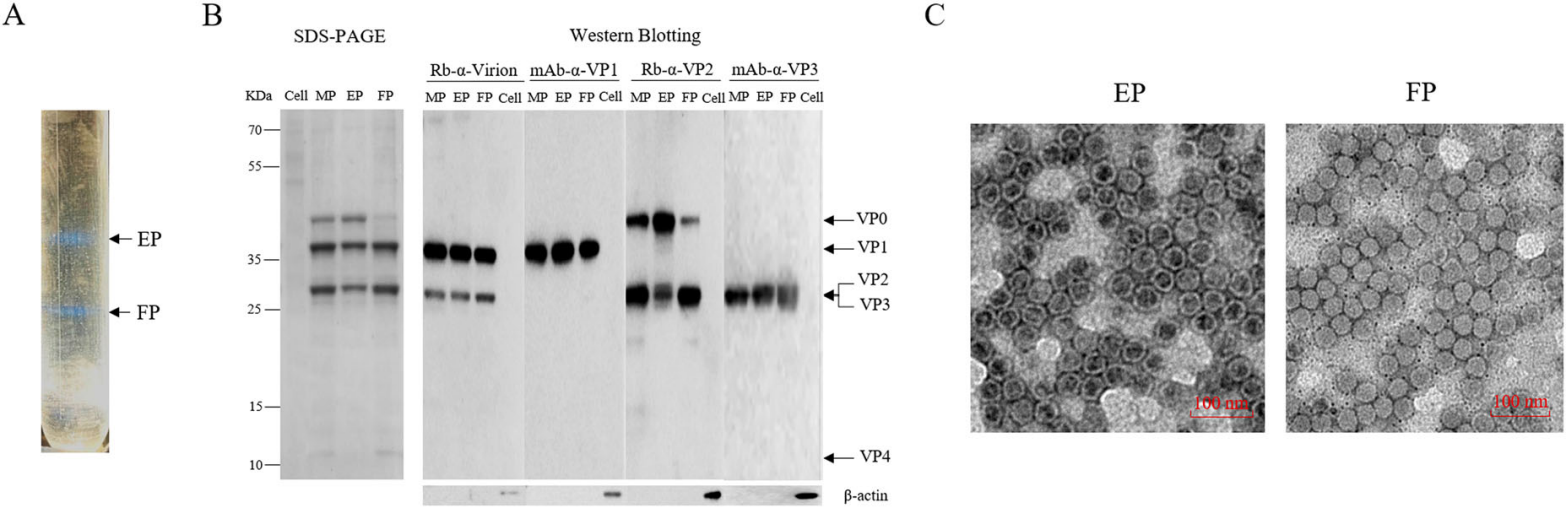
Purification and characterization of CV-A10 particles. CV-A10 particles were harvested from infected Vero cells, purified by CsCl gradient ultracentrifugation, and inactivated by formaldehyde as described in the materials and methods. (A) The positions of empty and full particles (EP, FP) were indicated in CsCl gradient (equilibrium at a starting density of 1.30 g per ml). The density of EP and FP particles was measured by an Ernst Abbe refractometer. (B) The purified particles EP, FP and their mixture MP (EP: FP = 4:6) were subjected to SDS-PAGE and Western blotting assays with rabbit polyclonal antibodies against whole virus, VP2 and mouse monoclonal antibody against VP1, VP3 (Rb-α-virion, mAb-α-VP1, Rb-α-VP2 and mAb-α-VP3). VP0, VP1, VP2, VP3 and VP4 were labelled on the right, and the molecular weight markers in KDa were indicated on the left. Mouse antibody against β-actin was used as loading control. (C) Electron microscope of inactivated EP and FP particles. Scale bar, 100 nm.

A RD-isolated and Vero-adapted strain, CV-A10-L12, was selected as a vaccine candidate. CV-A10-L12 was inoculated in a 10-layer cell factory of Vero cells at a multiplicity of infection (MOI) of 0.001. The virus harvest was purified by two steps of ultracentrifugation as described previously [26]. After ultracentrifugation, two white milky bands designated EPs (1.29 g/ml) and FPs (1.32 g/ml) were exhibited in CsCl gradient, as shown in Figure 1(A). CV-A10 protein profiles were analysed by SDS-PAGE and Western blotting with specific antibodies against whole virus, VP1, VP2 and VP3 (Figure 1(B)). MPs were a mixture component containing EPs and FPs at a natural particle ratio of 4:6, which represented the CV-A10 vaccine described below. EPs were composed of VP0, VP1 and VP3 proteins. FPs were consisted of VP1, VP2, VP3 and VP4 proteins. The identities of these structural proteins were confirmed by Western blotting using specific antibodies. As VP2 and VP3 proteins have similar molecular weight, these two proteins could not separate obviously in electrophoresis but proved comigrating by specific antibodies against the two viral proteins (Figure 1(B)). It is worth noting that the FP fraction contained a small quantity of VP0, which suggested that the composition of CV-A10 virion was the same as the polioviruses showed in the previous study [29]. Phillips et al. reported that mature virion of poliovirus was composed of 60 copies of structural proteins VP1 and VP3, and 58 or 59 copies of proteins VP2 and VP4, as well as 1 or 2 copies of uncleaved precursor VP0. The identities of EP and FP were further confirmed by TEM examination (Figure 1(C)), indicating CsCl ions penetrated the loose particles of EP.

### Humoral immunogenicity and immune persistence of inactivated CV-A10 vaccine

**Figure 2.**
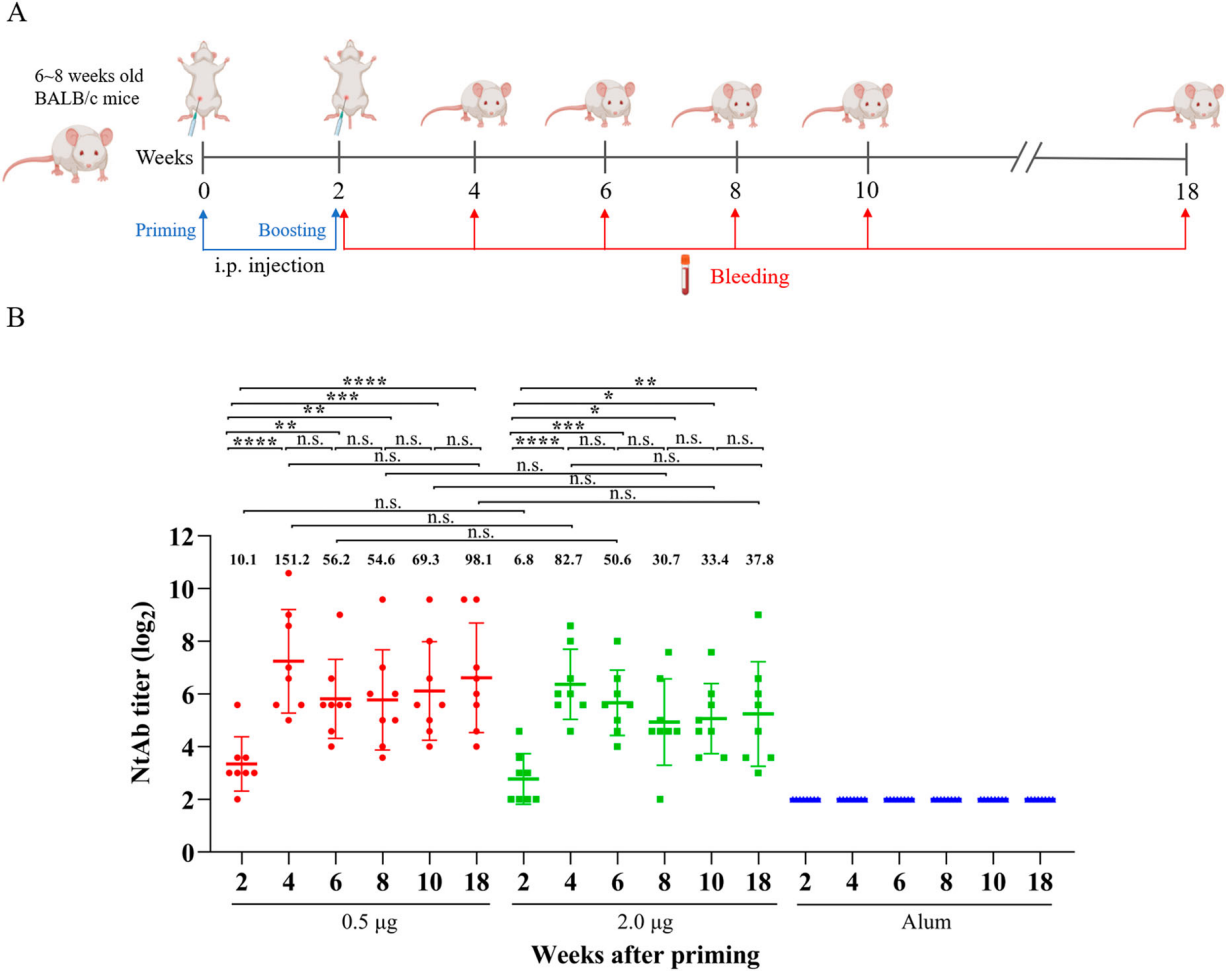
Humoral immune response and persistence of the inactivated CV-A10 vaccine. (A) The animal experiment was performed following the procedures explained in the schematic diagram. Groups of 6∼8 weeks old BALB/c mice were injected intraperitoneally with the inactivated CV-A10 vaccine on weeks 0 and 2 at doses of 0.5 or 2.0 μg per mouse (*n* = 8 per group). Control group was inoculated with Alum. Bleeding was performed on weeks 2, 4, 6, 8, 10 and 18, and the *in vitro* neutralizing antibody (NtAb) assay of the sera were performed. (B) NtAb levels were presented as the geometric mean titre (GMT) ± the standard error of the mean (SEM). Each symbol represented a mouse and the solid line indicated the GMT of the group. The bold numbers above the symbols represented the GMT of each group. NtAb titres below 8 were assigned to 2 for the convenience of presentation. One-way ANOVA was used for analysis of statistical significance: n.s., not significant (*P* ≥ 0.05); *, 0.01 ≤ *P* < 0.05; **, *P *< 0.01; ***, *P* < 0.001 and ****, *P* < 0.0001.

The immunogenicity and immune persistence of the purified, inactivated CV-A10 vaccine were tested in 6∼8-week-old BALB/c female mice as shown in Figure 2(A). The vaccines were inoculated on weeks 0, 2 and the sera from immunized mice were collected on weeks 2, 4, 6, 8, 10 and 18 post-priming. The micro-neutralization assay was performed to determine the capacity of the sera to neutralize the homologous strain CV-A10-L12. As shown in Figure 2(B), the geometric mean titre (GMT) of sera from the vaccine groups reached the peaks (0.5 µg: 151.2, 2.0 µg: 82.7) on week 4 post-priming. Thereafter, neutralizing antibodies maintained at a relatively stable level from 6 to 18 weeks post-priming, indicative of excellent immune persistence, considering the average 2-year lifetime of mice. No neutralization activity was detected in the control group even at the lowest dilution (1:8), suggesting that the induced humoral immunity was CV-A10 specific. Boosting significantly increased levels of NtAb at both low (0.5 µg) and high (2.0 µg) doses. However, there were no differences in NtAb levels in an antigen range from 0.5 to 2.0 µg. The results demonstrated that the inactivated CV-A10 vaccine had a good immunogenicity and immune persistence.

### Cellular immune response in CV-A10 immunized mice against VP1 and VP3 peptides

**Figure 3.**
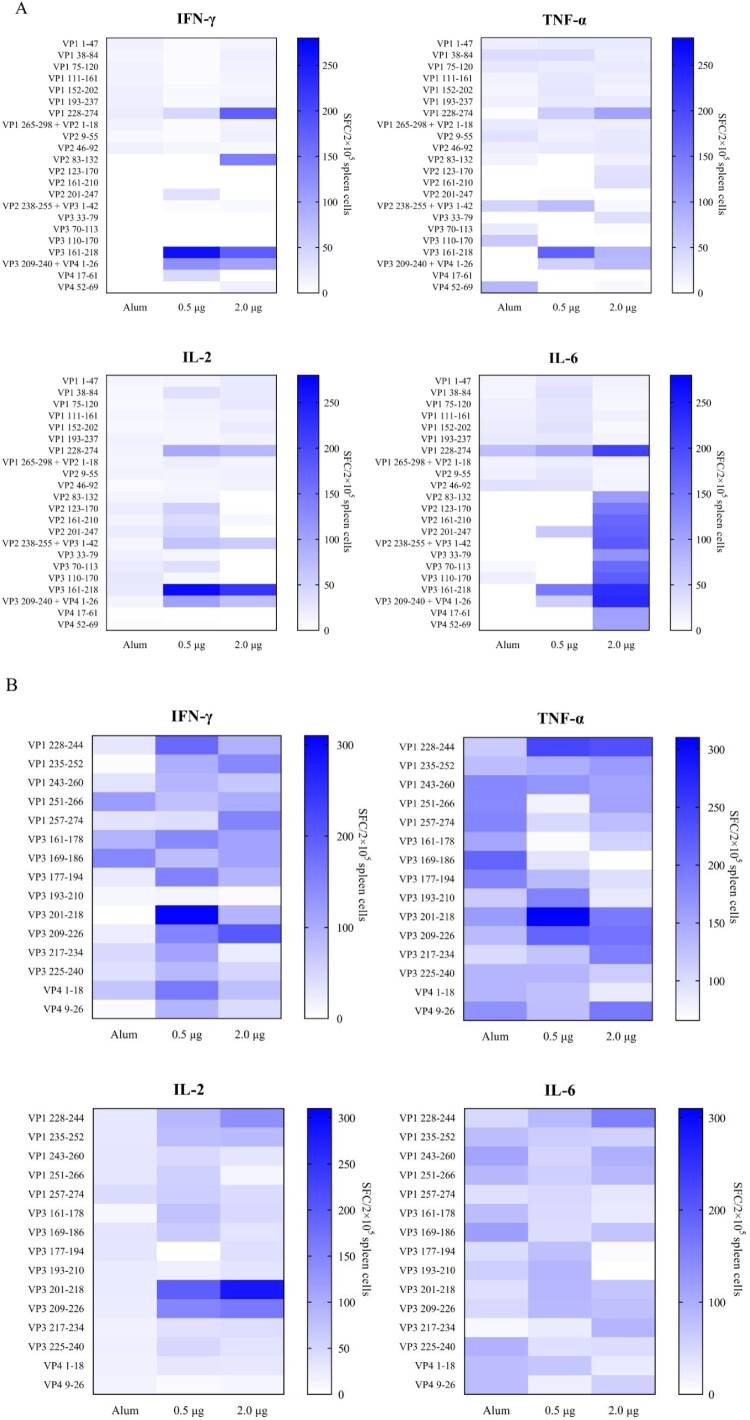
Specificity of proliferative T cell responses following CV-A10 immunization. Spleen cells of three BALB/c mice from groups were isolated for ELISPOT assays 14 days after secondary immunization with the inactivated CV-A10 vaccine. Proliferation was measured and the spot-forming cells (SFC) of spleen cells were counted 36 h after stimulation with synthetic peptides spanning the whole capsid protein. (A) Five adjacent peptides were mixed together to constitute a peptide pool, except for the single peptide VP4-(aa 52–69). (B) the peptide pools which could elicit strong positive T cell response were separated into single peptide. The selection criteria for positive responses were that the mean of detected SFC was ≥ 50 SFC per 2 × 10^5^ input cells.

**Figure 4.**
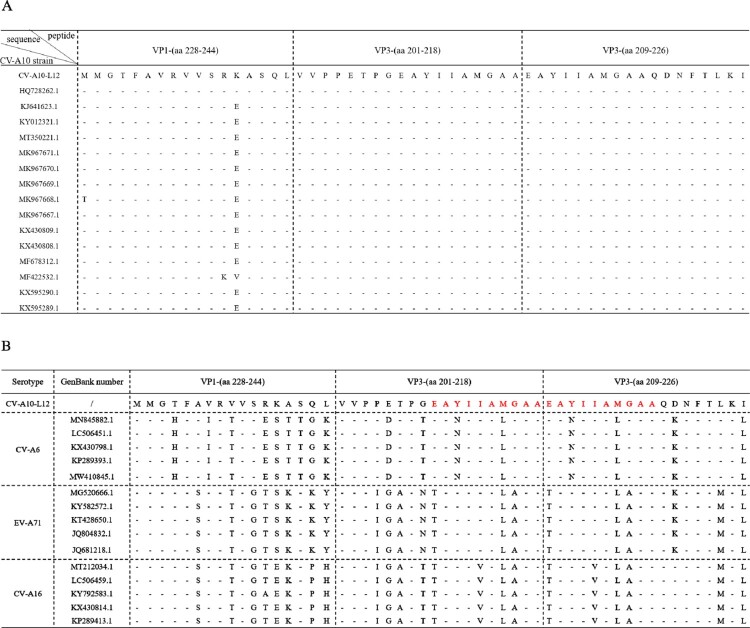
Alignment analysis of dominant epitopes among human enteroviruses. (A) Three identified T cell epitopes were highly conserved within circulating CV-A10 isolates. (B) The sequence of three epitopes showed obvious variation among other major circulating enteroviruses. The red amino acid sequences indicated the overlapping region between VP3-(aa 201–218) and VP3-(aa 209–226).

To evaluate cellular immune responses against CV-A10 in vaccine-inoculated BALB/c mice, we synthesized overlapping peptides spanning the entire sequence of the four structural proteins of CV-A10-L12. Unfortunately, four peptides were not successfully synthesized, including VP3-(aa 104–119), VP3-(aa 116–133), VP3-(aa 148–165) and VP3-(aa 185–202). Firstly, five adjacent peptides (overlapped by 10 aa for each other) were mixed to form a peptide pool. Freshly isolated spleen lymphocyte cells from the mice of three groups (0.5, 2.0 µg and Alum, 3 mice per group) were separately stimulated with the peptide pools. T cell responses were determined by *ex vivo* ELISPOT assays and analysed as SFCs per 2 × 10^5^ spleen cells. Three peptide pools elicited dominant positive responses resided in VP1, VP3 and VP4 (Figure 3(A)). The first one was VP1-(aa 228–274), which located at the C-terminal pool of VP1. The second one was VP3-(aa 161–218), which located at the C-terminal pools of VP3. The third one consisted of VP3-(aa 209–240) and VP4-(aa 1–26). Then these three peptide pools were analysed individually for further epitope-mapping. As shown in Figure 3(B), VP1-(aa 228–244), VP3-(aa 201–218) and VP3-(aa 209–226) were identified as the T-cell peptides for stimulating secretion of IFN-γ, TNF-α, IL-2 and IL-6. Interestingly, VP3-(aa 201–218) and VP3-(aa 209–226) had an overlapping by 10 aa. To investigate the sequence conservation of CV-A10 T cell epitopes among circulating CV-A10 isolates, the amino acid sequences corresponding to three identified peptides in 15 CV-A10 strains were aligned. As shown in Figure 4(A), all three sequences maintained high conservation in CV-A10 isolates. We also wondered whether other major causative pathogens of HFMD had the sequence homology corresponding to the amino acid sequences of these three peptides. The amino acid sequences of CV-A6, EV-A71 and CV-A16 strains were aligned as shown in Figure 4(B). The VP1-(aa 228-244), VP3-(aa 201-218) and VP3-(aa 209-226) were not conserved among co-circulating enteroviruses. Altogether, the regions VP1-(aa 228-244), VP3-(aa 201-218) and VP3-(aa 209-226) contained CV-A10 specific T-cell epitopes, and dominated T cell responses against CV-A10 in vaccinated BALB/c mice.

### 14-day-old Kunming mice are susceptible to CV-A10-M14 infection

**Figure 5.**
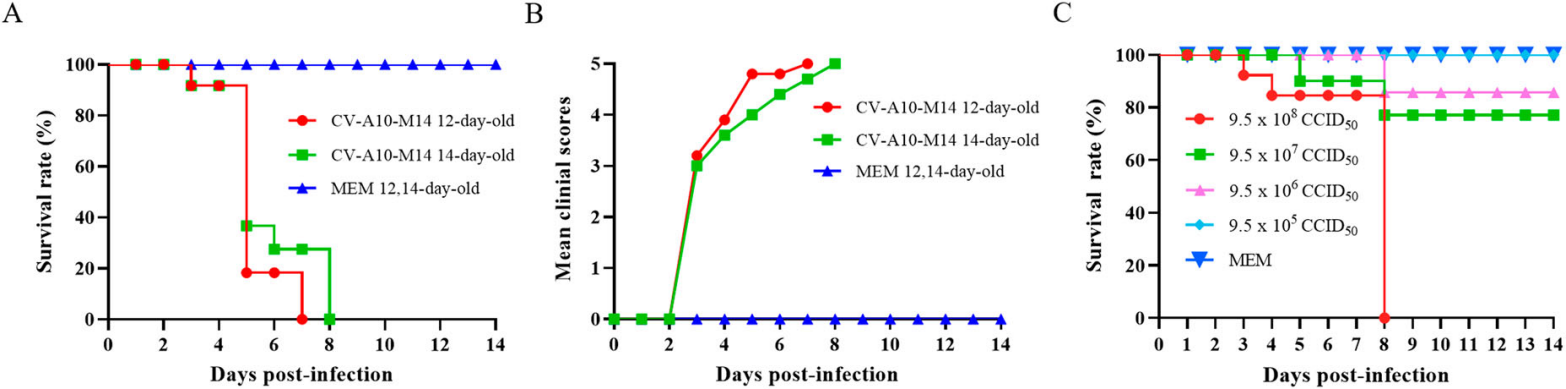
CV-A10-M14 susceptible Kunming mouse model. Kunming mice at 12 and 14 days of age were intraperitoneally (i.p.) inoculated with CV-A10-M14 at dose of 9.5 × 10^8^ CCID_50_/mouse. Control groups were inoculated with MEM medium. Survival rate (A) and clinical scores (B) were monitored and recorded daily until 14 days post-infection (dpi). The LD_50_ of CV-A10-M14 was determined through the i.p. inoculation at the doses ranging from 9.5 × 10^5^ to 9.5 × 10^8^ CCID_50_/mouse (C).

To determine the susceptibility of old age mice to a mouse-adapted strain, 10 neonatal Kunming mice at age of 12 and 14 days were i.p. infected with CV-A10-M14 at a dose of 9.5 × 10^8^ CCID_50_ per mouse in 300 μl. Clinical symptoms and survival rate were monitored daily after infection. As shown in Figure 5(A,B), 12-day-old and 14-day-old mice infected with CV-A10-M14 exhibited limb paralysis and ultimately all died at 7 or 8 dpi, respectively. In contrast, no clinical symptoms and death were observed in the MEM-inoculated mice at age of 12 and 14 days.

To further investigate the effect of infective dose on mobility and mortality, 14-day-old Kunming mice were i.p. challenged with 9.5 × 10^5^–9.5 × 10^8^ CCID_50_/300 μl/mouse (10-fold serially diluted) of CV-A10-M14. Mice i.p. inoculated with 9.5 × 10^8^ CCID_50_ began to die at 3 dpi and eventually all died within 8 dpi. With the inoculated doses of 9.5 × 10^7^ and 9.5 × 10^6^ CCID_50_, the survival rates were 77.1% and 85.7%, respectively (Figure 5C). In contrast, no death was observed in the mice inoculated with 9.5 × 10^5^ CCID_50_ and MEM. Therefore, the morbidity, course of symptoms and survival rates correlated well with the infective doses, and the LD_50_ was calculated as 1.9 × 10^8^ CCID_50_/mouse. The results demonstrated that CV-A10-M14 inoculated via i.p. route with a higher dose could develop severe symptoms and death of 14-day-old mice. Hence, a dose of 4.8 × 10^9^ CCID_50_/mouse (∼25 LD_50_) was optimized for subsequent active immunization and challenge experiments.

### Efficacy of the CV-A10 vaccine candidate in Kunming mice

**Figure 6.**
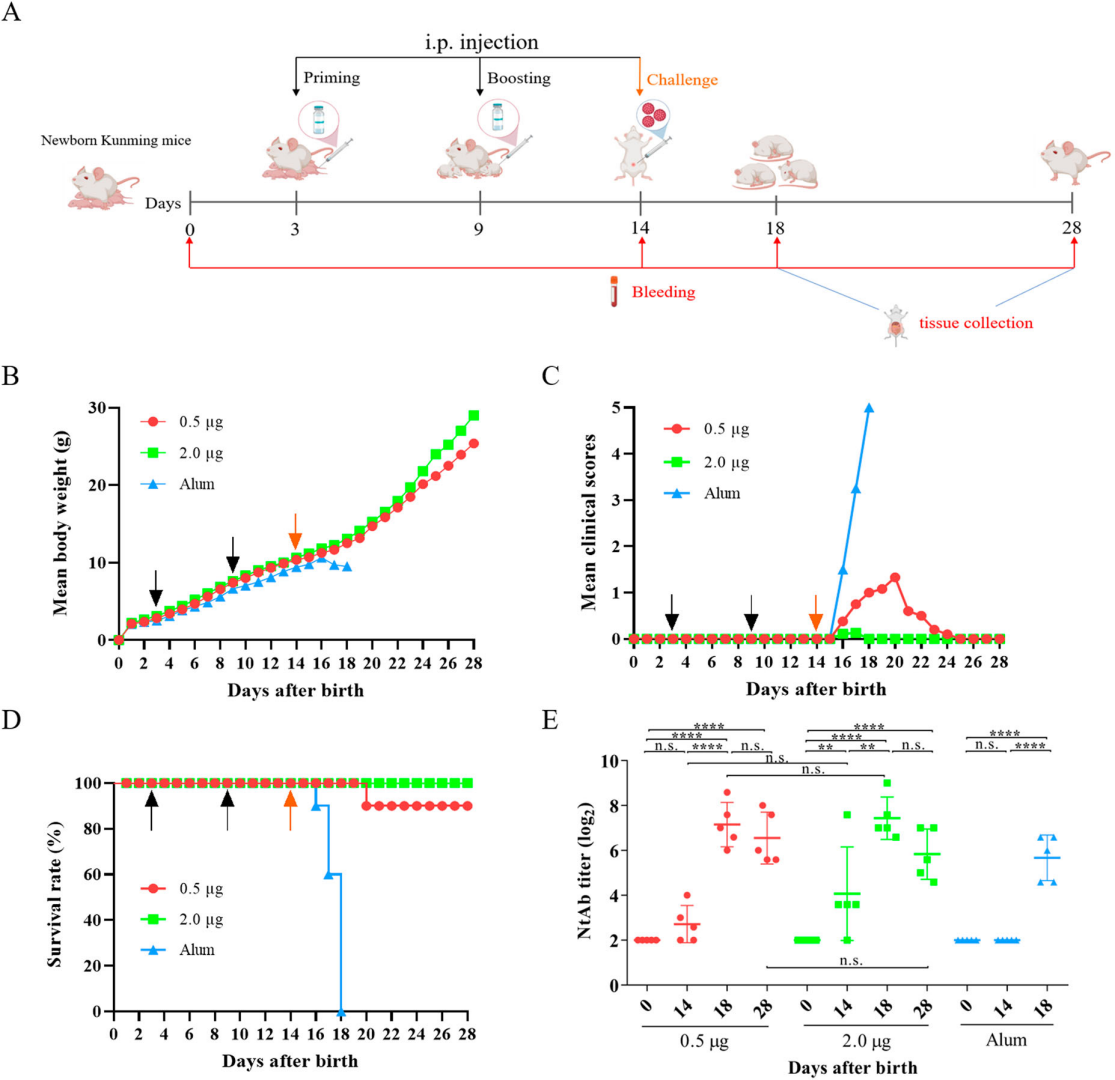
Protective efficacy of inactivated CV-A10 vaccine in the CV-A10 active immunized-challenged mouse model. (A) The animal experiment was performed as the schematic diagram showed. Kunming mice (*n* = 10) were primed and boosted via i.p. route at low or high dose (0.5 or 2.0 μg/mouse) on days 3 and 9, and challenged at a dose of 25 LD_50_ (4.8 × 10^9^ CCID_50_/mouse) on day 14. Control groups were immunized with Alum. The dates of vaccination and challenge were indicated by black and orange arrows, respectively. Bleeding (red arrows) was performed on days 0, 14, 18 and 28. The Alum-inoculated mice were euthanized before dying on day 18 to collect the sera. Tissues collection (blue lines) was performed on days 18 and 28. The body weight changes (B), clinical scores (C) and survival rate (D) were monitored daily until 14 days post-infection. (E) NtAb titres of the sera collected at different time points (*n* = 5 for each group) were determined and presented as the GMT (the solid line) ± SEM. For the convenience of figure presentation, NtAb titres below 8 were assigned to 2. Each symbol represented a mouse. **, **** and n.s., indicating *P* < 0.01, *P* < 0.0001 and no significant difference, respectively.

The *in vivo* protective efficacy of the inactivated CV-A10 vaccines was further evaluated by active immunization/challenge assay (Figure 6(A)). Groups of newborn Kunming mice were i.p. primed with 0.5 and 2.0 µg of the inactivated CV-A10 vaccine or the Alum control on day 3 and boosted on day 9. Five days later, the mice were challenged with CV-A10-M14 at a dose of 4.8 × 10^9^ CCID_50_/mouse. As shown in Figure 6(B,C), the mice were well tolerated to the vaccine because there were no significant differences in body weight changes or clinical scores between the vaccine-immunized and Alum-inoculated mice before challenge. After challenge, body weight decreased and clinical scores increased quickly for Alum-inoculated mice, compared with the mice in two immunized groups. The mice immunized with Alum developed severe clinical symptoms including retardation, limb weakness and paralysis during the course of observation, and all of them died at 4 days post-infection (Figure 6(C,D)). In contrast, the mice immunized with 2.0 µg of inactivated vaccine were free of clinical symptoms and were fully protected from death during the 14-day observation period. The mice immunized with 0.5 µg vaccine showed minor symptoms (reduced mobility) and the survival rate was 90%. Only one mouse exhibited limb paralysis and resulted in death at 6 dpi (Figure 6(D)). The protection of mice from disease and death was correlated to the vaccination in a dose-dependent manner.

To investigate the neutralizing antibody responses in mice, the sera from groups were tested for NtAb titres on days 0, 14, 18 and 28 (Figure 6(E)). These sera represented samples taken at the time of pre-immunization, pre-challenge, post-challenge and end of observation period (Figure 6(A)). The Alum-inoculated mice were euthanized before dying to collect their sera on day 18. As shown in Figure 6(E), neutralizing antibodies were detectable in mice after boosting on day 14, and the sera conversion rate for mice inoculated with 0.5 and 2.0 µg vaccines were 60% and 80%, respectively, in a dose-dependent way. After challenge, a high NtAb titre was exhibited in 18-day-old mice immunized with two doses of 0.5 or 2.0 µg vaccines, whereas the NtAb titre in the mice injected with Alum was lower than those of two immunized groups (0.5 µg: 141.8, 2.0 µg: 172.7, Alum: 50.7). The results indicated a rapid immune response following challenge. Furthermore, there was no significant differences of NtAb titres between days 18 and 28 in each group of the vaccine-immunized mice. Meanwhile, no obvious dose-dependent effect was showed in NtAb titres between 0.5 and 2.0 µg groups. These results demonstrated that the inactivated CV-A10 vaccine could elicit a strong humoral immune response, together with cellular immune response described above, to protect mice from disease and death caused by CV-A10 infection.

### Viral loads in organs of CV-A10 immunized-challenged mice

**Figure 7.**
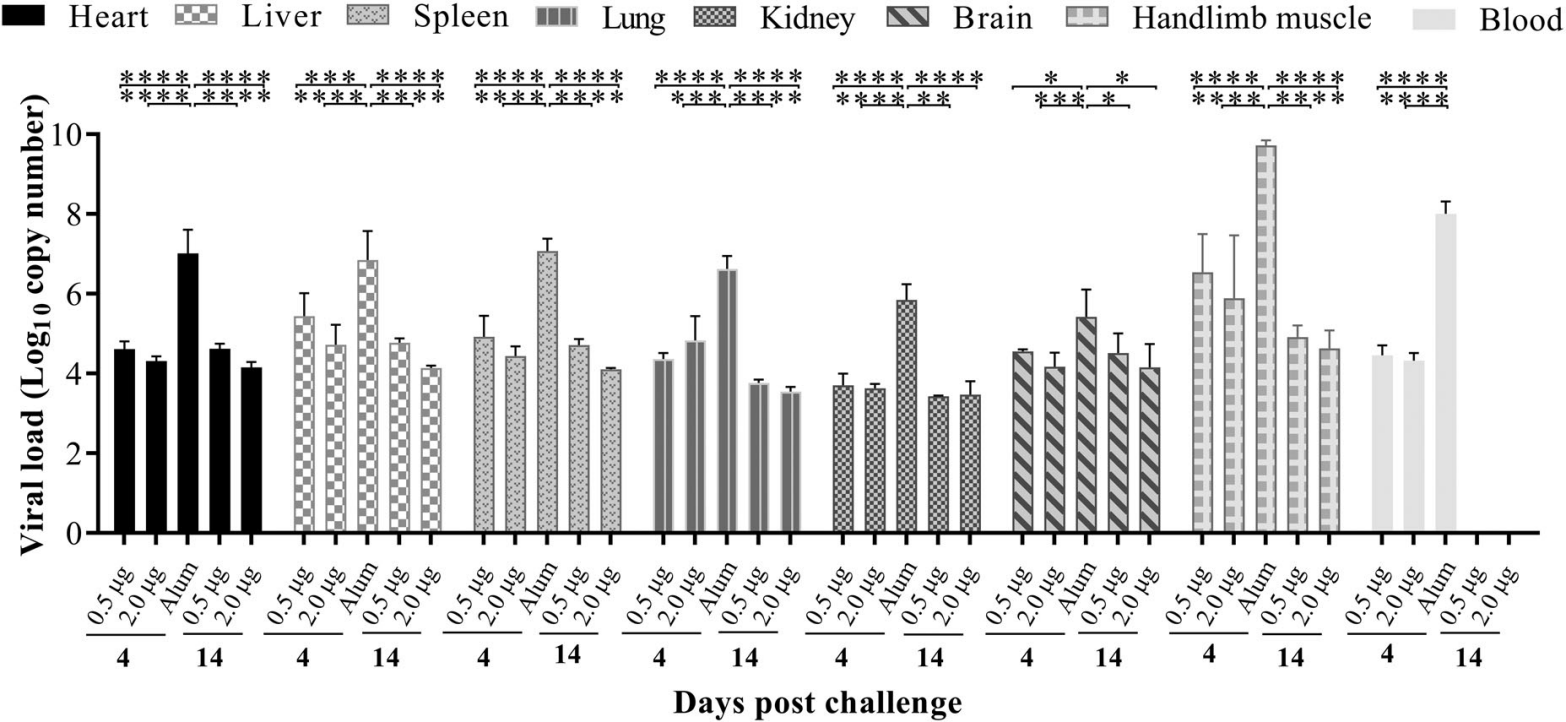
Tissue virus loads in immunized and challenged Kunming mice. Kunming mice inoculated with 0.5, 2.0 μg vaccines or Alum were infected with 25 LD_50_ (4.8 × 10^9^ CCID_50_/mouse) of CV-A10-M14. To detect the viral load *via* qRT-PCR, heart, liver, spleen, lung, kidney, brain, intestine, blood and hindlimb muscle tissues were collected from the mice at 4 and 14 day-post-challenge. Virus loads were quantified by a standard curve obtained from 10-fold serial dilutions of CV-A10 transcript. The data represent the mean ± SEM for five mice per group and were analysed with one-way ANOVA (*, 0.01 ≤ *P* < 0.05; **, *P* < 0.01; ***, *P* < 0.001; ****, *P *< 0.0001).

To understand the dynamics of viral loads in CV-A10 immunized-challenged mice, blood and different tissue samples were analysed by qRT-PCR on days 4 and 14 post-challenge. In Alum-inoculated mice, the highest amount of the CV-A10 RNA detected was in the hindlimb muscle, followed by those in the blood, spleen, heart, liver, lung, kidney, brain at 4 days post-challenge (Figure 7). Compared with high viral replication in Alum-inoculated mice, viral RNA copies could be detected at relatively low levels in all organs and blood collected from vaccinated mice at 4 days post-challenge. A viral load was hardly detected in the intestines of all tested mice throughout the experiment (data not shown) and in the blood at 14 days post-challenge. The viral loads showed an obvious downward trend in most organs from two vaccinated groups at 14 days post-challenge. These findings indicated that CV-A10 showed a strong tropism to the hindlimb muscle of neonatal Kunming mice. Detection of viral RNA in brain and blood suggested that CV-A10 could cause viremic spread and invade into central never system.

### Histopathologic and immunohistochemistry (IHC) analysis

**Figure 8.**
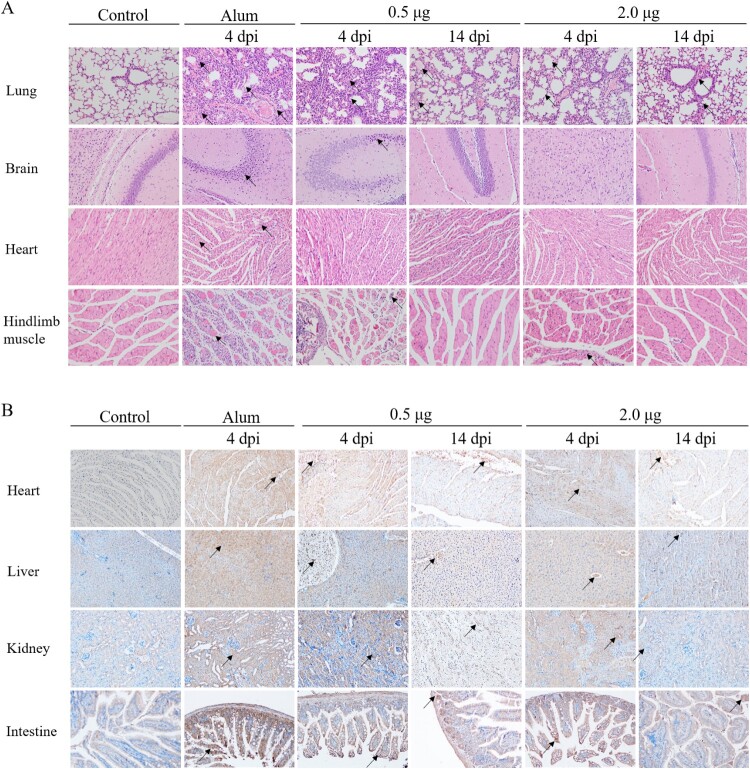
The analysis of the histopathological (H&E) and immunohistochemical (IHC) examination of tissues from immunized-challenged mice (200×). Immunized Kunming mice were inoculated i.p. with CV-A10-M14. The Alum groups were euthanized at 4 days post-challenge, and mice in the vaccinated groups were euthanized at 4 and 14 day-post-challenge. The healthy Kunming mice at the age of 28 days served as the negative control. For the convenience of figure presentation, “days post-challenge” was represented by “dpi.” (A) The lung, brain, heart and hindlimb muscle organs were sectioned and observed. (B) IHC assays for the organs of heart, liver, kidney and intestine were performed using a rabbit polyclonal antibody against CV-A10 whole virus. Black arrows indicated representative pathological damage (A) and expression of viral protein (B).

To further study pathogenesis and the mechanism of protection of vaccination, H&E and IHC staining were performed in vaccinated or Alum-inoculated mice (grade level above 4) on days 4 and 14 after CV-A10-M14 challenge. The heart, liver, spleen, lung, kidney, brain, intestines and hindlimb muscles were collected and analysed to compare the pathological changes and antigenic distribution between the vaccinated or Alum control mice. As shown in Figure 8(A), the H&E results showed that the lung and hindlimb muscle derived from Alum-inoculated mice exhibited cellular damage (such as degeneration and necrosis) and increased numbers of inflammatory cells. Severe pathological changes were also observed in the brain and heart tissue from Alum-inoculated mice. However, in the mice from two vaccinated groups, the lung, hindlimb and other tested organs or tissues showed mild pathological changes compared to Alum-inoculated mice. The black arrows indicated lesions of the tissues. Here, we chose some organs to describe the pathological changes. In the lung, the alveolar structure was destroyed and varied in shape or size, the tracheal epithelial cells were arranged in disorder, more connective tissue proliferating exhibited around the blood vessels, and the alveoli were filled with a large number of blood cells and inflammatory cells. In the hindlimb muscle, muscle fibres were divided and replaced by connective tissue, vacuolar degenerated severely, the partial atrophy of muscle fibres was observed, and increased numbers of inflammatory cells infiltrated between the muscle fibres. In the brain, large areas of neuronal nuclei were pyknotic and deeply stained. In the heart, myocardial fibre necrosed and hypochromatosis was observed.

In the IHC (Figure 8(B)) experiments, viral antigen, probed with anti-CV-A10 antibody, was detected in the lung, brain, spleen, heart, liver, intestine, kidney and hindlimb muscle from the Alum-inoculated group. The levels of viral protein expression of vaccinated mice in the above organs or tissues were lower than those in the Alum-inoculated mice at 4 days post-challenge, and decreased obviously at 14 days post-challenge (the end of the observation period). An IPP6.0 image analysis system was applied to calculate the mean optical density based on the positive area from IHC images (as shown in supplemental Figure S2). The mean optical density was consistent with the results of viral loads that the levels of viral protein in organs were higher in the Alum-inoculated group. Among the two vaccinated groups, antigen detected in mice immunized with 2.0 μg vaccine was lower than that of 0.5 μg vaccine at 4 days post-challenge. The H&E and IHC analysis demonstrated that the inactivated vaccine could protect the mice from the lethal challenge of CV-A10-M14 and cause mild symptoms in the mice.

## Discussion

Murine and non-primate models are the two major animal models used for evaluating vaccines against HFMD. As HFMD-related viruses only cause non-lethal infections in adult mice, the common method to evaluate the protective efficacy of experimental vaccines is a passive immunized mouse model, which was performed in two ways [3]. One way was to transfer antiserum derived from immunized adult mice to newborn suckling mice and to examine their effect on subsequent viral challenges [13,14,18,30,31]. The other way was recently developed by first combining immunized serum with virus *in vitro* and then applying the neutralization mixture to suckling mice followed by virus challenge [32]. However, these two strategies cannot truly reflect the cellular and humoral responses directly induced by vaccines in neonatal mice. Furthermore, the active immunization-challenge animal models for CV-A10 are less developed [15,17,21]. Hence, there is an urgent need to establish a mouse model suitable for evaluating active immunity for CV-A10.

In 2016, Shen et al. [17] first evaluated a whole CV-A10 inactivated vaccine on ICR mouse. The mice were i.p. injected with the inactivated vaccine on days 1 and 6, followed by a challenge with CV-A10 on day 12. The result showed that the vaccine conferred perfect protection against the homologous and heterologous infection in mice. However, the mechanism of vaccine protection and the characteristics of pathology caused by CV-A10 infection were not clear. In 2018, Zhang et al. [21] evaluated an inactivated, whole-virus bivalent (CV-A6 ad CV-A10) vaccine on ICR mice, based on their previous monovalent vaccine studies [13,30]. One-day-old mice inoculated twice with the inactivated vaccines on days 1 and 5, and then were exposed to lethal doses of CV-A10 on day 10. The survival rate was 80% in the vaccine-immunized group, which showed that the bivalent vaccine exhibited protective effects. In 2020, Chen et al. [15] first developed a CV-A10-infected gerbil model using a clinically isolated CV-A10 strain, CVA10-4. This model was employed to assess the protective efficacy of an inactivated CV-A10 vaccine. One-day-old gerbils were i.p. immunized with the inactivated CV-A10 vaccine on days 8 and 11, and were inoculated with CV-A10 on day 14. The result showed that this CV-A10 vaccine could fully protect 14-day-old gerbils against a heterologous lethal challenge of CV-A10.

In this study, we used a mouse-adapted strain (CV-A10-M14) to develop a CV-A10 infection mice model, and this model was found to be efficacious for vaccine assessment through active immunization-challenge experiment. Compared to other whole-inactivated CV-A10 vaccines that have been published, our method to develop vaccine was unique. The clinical isolates couldn’t grow in Vero cells directly, so we passaged it in RD cells, then adapted it to Vero cells. The Vero-adapted strain, CV-A10-L12, was used to prepare an inactivated vaccine for CV-A10 by reverse genetics approach. In our previous study, the same method was used to prepare the Vero cell-based inactivated CV-A2, CV-A5 and CV-A6 vaccine [24,26,27]. This strategy can be widely applied in the development of other HFMD vaccines where the viruses cannot be easily isolated and efficiently propagated in high titres in Vero and MRC5 cell lines [7].

In our laboratory, the parental challenge virus could infect 1- to 10-day-old ICR mice, but not infect 14-day-old mice via intracranial route (i.c.) in 30 µl. Our previous studies demonstrated that a mouse infection model was dependent on the age and species of mice, virulence of strain, inoculating route and dose [24,26,27]. Inoculation via i.p route in 300 µl using concentrated virus stock delivered higher dosage and was easy to administer. Meanwhile, we chose another outbred mouse (Kunming mice) for mouse infection experiment. This mouse strain and inoculation route of i.p. have been successfully used to establish the CV-A2, CV-A5 and CV-A6 infection models in our previous studies [24,26,27]. Our results showed that the challenge virus CV-A10-M14 at a high dose of 9.5 × 10^8^ CCID_50_/mouse (5 LD_50_) was lethal to 14-day-old Kunming mice or younger by i.p route. All infected mice exhibited severe symptoms such as hindlimb paralysis and died within 8 days. Further investigation on the susceptibility of Kunming mice to CV-A10-M14 infection via intracerebral (i.c.), intramuscular (i.m.) and intraoral (i.o.) routes will be performed. A work from Chen et al. showed that the disease symptom of gerbils inoculated via the i.o. route was not obvious [15], which was in agreement with a previous study reported by Gao et al. [16]. The results of Chen et al. indicated that both the i.p. and i.m. routes were suitable for CV-A10 infection.

Similar to other enterovirus infection models, hindlimb muscle exhibited severe damage and had the highest viral titres in mice inoculated with Alum and challenged by CV-A10-M14 at 4 days post-challenge. Our results indicated that muscle was the most active site of viral replication, which was similar to that previously reported for EV-A71 and CV-A16 [33,34]. The severe pathological changes and the strong specific staining of tissue sections could also be observed in the lung, brain and heart. Meanwhile, viral loads in blood were present at a relatively higher level than those in other tissues, except for hindlimb muscles, which was consistent with the phenomena observed from the CV-A10 or CV-B1 infection model developed by Chen et al. or Yin et al., respectively [15,35]. These results suggested that CV-A10 entered the bloodstream, propagated in epithelial cells of pharynges and intestines [36], caused viremia and widely spread to various target organs for replication such as muscle, lung and heart. Viraemia spread might be one of the reasons to cause death in CV-A10-infected mice. In addition, the relatively high viral load and the appearance of viral antigens in the brain of Kunming mice indicated that CV-A10 exhibited active infection or tropism in the CNS of mice. However, the results from Chen et al. suggested that the CNS was not the major target tissue for CV-A10 replication because no obvious lymphocytic inflammatory infiltration or disorder of nerve cells in the brain was observed and the limb paralysis may, to a large extent, be due to the muscle damage rather than CNS tropism, which was consistent with a previous finding of Li et al. and Gao et al. [14,16]. In addition, a CV-A2 murine infection model established by Ji showed that high viral load was only detected in skeletal muscles at 3 dpi, and then viral replication was detected and reached the maximum at 7 dpi in other multiple organs [37]. Given the dynamic changes of viral load, Ji et al. questioned that whether limb paralysis was caused primarily by skeletal muscle or CNS damage, which needed to be determined. As we know, skeletal muscles support persistent enterovirus infection and provide a viral source of entry into the CNS during poliovirus infection [38]. It is worth noting that no viral RNAs were detected in the intestines, although the intestines were the primary infection site of enteroviruses. This phenomenon was also reported in our previous study [26]. It could be explained for two reasons. First, the virus in the intestines of mice which were dying may spread to other target organs, such as brain, heart and lung, at 4 days after challenge. If we collected the sample at one to four day-post-infection (dpi), viral RNAs may be detected in the intestines. Second, the virus has not existed in the site we sampled because the intestine was long.

Neutralizing antibodies are regarded as a mostly related correlator of protective immunity induced by vaccines. Some reports have demonstrated that neutralizing titres above 1:8 were sufficient to provide protective immunity, despite the antibody titres being relatively low [39,40]. The immunogenicity of the vaccine candidate, CV-A10-L12, was evaluated in BALB/c mice in our study. The inactivated vaccines at 0.5 µg/dose or 2.0 µg/dose were all capable of eliciting CV-A10 specific immune response upon immunization in mice. By *in vitro* neutralization assay, the inactivated CV-A10-L12 immunized sera showed the highest neutralizing titres against homologous virus at 28 days post-priming. Thereafter, neutralizing titres decreased slowly and remained stable, suggesting that CV-A10-L12 vaccine candidate had a good immune persistence. A similar study was also performed by Chen et al., but the results demonstrated that the inactivated CVA10-TZ3-P5 vaccine could not provide long-term protection, as the neutralizing antibodies significantly declined to 5.46 at the second-week post-immunization [15]. We speculated that this difference in immunogenicity of two vaccine candidates might be attributed to the immunogenicity of the virus strains, immunization doses and immunization interval.

Although the humoral immune responses to enteroviral infections have been well characterized, the nature, specificity and the functional role of the enterovirus T cell-specific response is less clarified so far. Studies in rodent have been carried out to identify T-cell epitopes on four structural proteins (VP1-VP4) of enteroviruses [41–43]. Several studies in humans were also performed using peripheral blood mononuclear cells (PBMCs) from adult donors [44–47]. Th cells, particularly Th1 and Th2 cells, play a major role in humoral and cellular immune responses. Th1 cells induce the cytokines interleukin 2 (IL-2), interferon gamma (IFN-γ) and tumour necrosis factor alpha (TNF-α), which are capable of promoting T-cell cytotoxicity. Th2 cells have the capacity to produce IL-4, IL-5, IL-6, IL-10 and IL-13, which enhance the B-cell response.

In this study, cytokine analysis showed that several cytokines, including IL-2, IL-6, IFN-γ and TNF-α, which are associated with Th1 and Th2 cells, were elevated in the two vaccinated groups. By using the synthetic peptides, strong proliferative responses were induced in BALB/c mice and three Th epitopes were identified, which were located at the carboxyl-terminal region of the capsid protein VP1 and VP3. One region surrounded residues 228–244 of VP1, a second region lay in residues 201–218 of VP3. A third similar Th epitope, which was reported as A8 to elicit high immune responses in human [45], was identified in the region from 209 to 226 of VP3. Alignment of the amino sequences showed that the three dominant peptides were highly conserved within CV-A10 strains, and revealed variation among the different but closely related species A enteroviruses. Interestingly, the regions from 201 to 218 and 209 to 226 of VP3 were overlapped at the ten-amino-acid sequence EAYIIAMGAA. Thus, we hypothesized that serotype-specific Th epitope in BALB/c mice for CV-A10 was mainly located within the ten-amino-acid sequence EAYIIAMGAA. However, when a previously identified EV-A71 Th epitope in humans, VP2-19 (aa 139–155), was tested, it failed to stimulate a proliferative response in CV-A10 immunized mice for our study (Data not shown) [46]. Besides, the inability of peptides representing epitopes in VP4 to induce lymphocyte stimulation was unexpected. In previous work, murine T cell clones which proliferated in response to all three poliovirus serotypes recognized VP4 [43]. These discrepancies in results between us and other researchers might be due to various reasons. First, findings in rodent models might not be consistent with the T cell response in humans infecting enteroviruses. Second, the differences observed may be caused by the conditions of the stimulation tests. Third, the sequences of synthetic peptides used by us were similar but not completely conserved, compared to those studied in previous works. Changes in single amino acid or different peptide chain length might affect the T cell recognition and abolish the T cell activation. To our knowledge, this study constitutes the first description to identify CV-A10 specific T cell epitopes using spleen cells from immunized mice, and provided solid evidence for a possible role for VP1 and VP3 in immunity to CV-A10. Analysis by Flow cytometry will be urgent needed and helpful for further study to measure the proportion of the polyfunctional CD4^+^ or CD8^+^ T cells expressing IFN-γ, TNF-α, IL-2 and IL-6.

In conclusion, a Vero-adapted strain was selected to prepare the CV-A10 inactivated vaccine, and the humoral and cellular immunogenicity analysis showed that inactivated CV-A10-L12 vaccine could elicit strong neutralizing antibody responses and T cell response in BALB/c mice. Meanwhile, a mouse-adapted strain was employed to establish a mouse model of CV-A10 infection and develop a CV-A10 active immunization/challenge mouse model for evaluating the protective efficacies of vaccines. Our study showed that the inactivated CV-A10 vaccines in different doses were capable of conferring protection against lethal CV-A10-M14 infection in the 14-day-old Kunming mouse. Pathological observations, IHC staining and viral loads assay revealed that the CV-A10-M14 strain had a strong tropism to hindlimb muscle tissues, causing severe necrosis and paralysis. This mouse model will provide valuable information for a comprehensive understanding of CV-A10 and the development of vaccines or drugs against CV-A10.

## Supplementary Material

Supplemental Material
