# Supplementary material for: Humoral and cellular immunogenicity and efficacy of a coxsackievirus A10 vaccine in mice
Source: Emerg Microbes Infect. 2023 Jan 17;12(1):e2147022. doi: 10.1080/22221751.2022.2147022 (PMC9848378; doi:10.1080/22221751.2022.2147022)
Supplement: Supplemental Material [file TEMI_A_2147022_SM3864.doc]

Supplemental data

Table S1. Overlapping peptides used in the present study

| Peptide name | Sequence | Length  (amino acids) |
| --- | --- | --- |
| CVA10 VP4-1 (1-18)^a^ | MGAQVSTQKSGSHETGNV | 18 |
| CVA10 VP4-2 (9-26) | KSGSHETGNVATGGSTIN | 18 |
| CVA10 VP4-3 (17-34) | NVATGGSTINFTNINYYK | 18 |
| CVA10 VP4-4 (25-41) | INFTNINYYKDSYAASA | 17 |
| CVA10 VP4-5 (32-47) | YYKDSYAASATRQDFT | 16 |
| CVA10 VP4-6 (38-54) | AASATRQDFTQDPKKFT | 17 |
| CVA10 VP4-7 (45-61) | DFTQDPKKFTQPVLDSI | 17 |
| CVA10 VP4-8 (52-69) | KFTQPVLDSIRELSAPLN | 18 |
| CVA10 VP2-1 (1-18) | SPSVEACGYSDRVAQLTV | 18 |
| CVA10 VP2-2 (9-23) | YSDRVAQLTVGNSSI | 15 |
| CVA10 VP2-3 (14-31) | AQLTVGNSSITTQEAANI | 18 |
| CVA10 VP2-4 (22-39) | SITTQEAANIVLAYGEWP | 18 |
| CVA10 VP2-5 (30-47) | NIVLAYGEWPEYCPDTDA | 18 |
| CVA10 VP2-6 (38-55) | WPEYCPDTDATAVDKPTR | 18 |
| CVA10 VP2-7 (46-63) | DATAVDKPTRPDVSVNRF | 18 |
| CVA10 VP2-8 (54-71) | TRPDVSVNRFYTLDSKMW | 18 |
| CVA10 VP2-9 (62-79) | RFYTLDSKMWQENSTGWY | 18 |
| CVA10 VP2-10 (70-86) | MWQENSTGWYWKFPDVL | 17 |
| CVA10 VP2-11 (77-92) | GWYWKFPDVLNKTGVF | 16 |
| CVA10 VP2-12 (83-100) | PDVLNKTGVFGQNAQFHY | 18 |
| CVA10 VP2-13 (91-108) | VFGQNAQFHYLYRSGFCL | 18 |
| CVA10 VP2-14 (99-116) | HYLYRSGFCLHVQCNASK | 18 |
| CVA10 VP2-15 (107-124) | CLHVQCNASKFHQGALLV | 18 |
| CVA10 VP2-16 (115-132) | SKFHQGALLVAVIPEFVI | 18 |
| CVA10 VP2-17 (123-140) | LVAVIPEFVIAGRGSNTK | 18 |
| CVA10 VP2-18 (131-148) | VIAGRGSNTKPNEAPHPG | 18 |
| CVA10 VP2-19 (139-155) | TKPNEAPHPGFTTTFPG | 17 |
| CVA10 VP2-20 (146-162) | HPGFTTTFPGTTGATFH | 17 |
| CVA10 VP2-21 (153-170) | FPGTTGATFHDPYVLDSG | 18 |
| CVA10 VP2-22 (161-178) | FHDPYVLDSGVPLSQALI | 18 |
| CVA10 VP2-23 (169-186) | SGVPLSQALIYPHQWINL | 18 |
| CVA10 VP2-24 (177-194) | LIYPHQWINLRTNNCATV | 18 |
| CVA10 VP2-25 (185-202) | NLRTNNCATVIVPYINAV | 18 |
| CVA10 VP2-26 (193-210) | TVIVPYINAVPFDSAINH | 18 |
| CVA10 VP2-27 (201-218) | AVPFDSAINHSNFGLIVI | 18 |
| CVA10 VP2-28 (209-225) | NHSNFGLIVIPVSPLKY | 17 |
| CVA10 VP2-29 (216-233) | IVIPVSPLKYSSGATTAI | 18 |
| CVA10 VP2-30 (224-240) | KYSSGATTAIPITITIA | 17 |
| CVA10 VP2-31 (231-247) | TAIPITITIAPLNSEFG | 17 |
| CVA10 VP2-32 (238-255) | TIAPLNSEFGGLRQAVSQ | 18 |
| CVA10 VP3-1 (1-18) | GIPAELRPGTNQFLTTDD | 18 |
| CVA10 VP3-2 (9-25) | GTNQFLTTDDDTAAPIL | 17 |
| CVA10 VP3-3 (16-34) | TDDDTAAPILPGFTPTPTI | 19 |
| CVA10 VP3-4 (25-42) | LPGFTPTPTIHIPGEVHS | 18 |
| CVA10 VP3-5 (33-50) | TIHIPGEVHSLLELCRVE | 18 |
| CVA10 VP3-6 (41-58) | HSLLELCRVETILEVNNT | 18 |
| CVA10 VP3-7 (49-66) | VETILEVNNTTEATGLTR | 18 |
| CVA10 VP3-8 (57-73) | NTTEATGLTRLLIPVSS | 17 |
| CVA10 VP3-9 (64-79) | LTRLLIPVSSQNKADE | 16 |
| CVA10 VP3-10 (70-85) | PVSSQNKADELCAAFM | 16 |
| CVA10 VP3-11 (76-90) | KADELCAAFMVDPGR | 15 |
| CVA10 VP3-12 (81-98) | CAAFMVDPGRIGPWQSTL | 18 |
| CVA10 VP3-13 (89-106) | GRIGPWQSTLVGQICRYY | 18 |
| CVA10 VP3-14 (97-113) | TLVGQICRYYTQWSGSL | 17 |
| CVA10 VP3-16 (110-125) | SGSLKVTFMFTGSFMA | 16 |
| CVA10 VP3-18 (124-141) | MATGKMLVAYSPPGSAQP | 18 |
| CVA10 VP3-19 (132-149) | AYSPPGSAQPANRETAML | 18 |
| CVA10 VP3-20 (140-157) | QPANRETAMLGTHVIWDF | 18 |
| CVA10 VP3-22 (156-170) | DFGLQSSVSLVIPWI | 15 |
| CVA10 VP3-23 (161-178) | SSVSLVIPWISNTHFRTA | 18 |
| CVA10 VP3-24 (169-186) | WISNTHFRTAKTGGNYDY | 18 |
| CVA10 VP3-25 (177-194) | TAKTGGNYDYYTAGVVTL | 18 |
| CVA10 VP3-27 (193-210) | TLWYQTNYVVPPETPGEA | 18 |
| CVA10 VP3-28 (201-218) | VVPPETPGEAYIIAMGAA | 18 |
| CVA10 VP3-29 (209-226) | EAYIIAMGAAQDNFTLKI | 18 |
| CVA10 VP3-30 (217-234) | AAQDNFTLKICKDTDEVT | 18 |
| CVA10 VP3-31 (225-240) | KICKDTDEVTQQAVLQ | 16 |
| CVA10 VP1-1 (1-18) | GDPVEDIIHDALGNTARR | 18 |
| CVA10 VP1-2 (9-26) | HDALGNTARRAISSVTNV | 18 |
| CVA10 VP1-3 (17-34) | RRAISSVTNVESAANTTP | 18 |
| CVA10 VP1-4 (25-39) | NVESAANTTPSSHRL | 15 |
| CVA10 VP1-5 (30-47) | ANTTPSSHRLETGRVPAL | 18 |
| CVA10 VP1-6 (38-55) | RLETGRVPALQAAETGAT | 18 |
| CVA10 VP1-7 (46-63) | ALQAAETGATSNATDENM | 18 |
| CVA10 VP1-8 (54-70) | ATSNATDENMIETRCVV | 17 |
| CVA10 VP1-9 (61-76) | ENMIETRCVVNRNGVL | 16 |
| CVA10 VP1-10 (67-84) | RCVVNRNGVLETTINHFF | 18 |
| CVA10 VP1-11 (75-90) | VLETTINHFFSRSGLV | 16 |
| CVA10 VP1-12 (81-98) | NHFFSRSGLVGVVNLTDG | 18 |
| CVA10 VP1-13 (89-105) | LVGVVNLTDGGTDTTGY | 18 |
| CVA10 VP1-14 (96-112) | TDGGTDTTGYATWDIDI | 17 |
| CVA10 VP1-15 (103-120) | TGYATWDIDIMGFVQLRR | 18 |
| CVA10 VP1-16 (111-128) | DIMGFVQLRRKCEMFTYM | 18 |
| CVA10 VP1-17 (119-136) | RRKCEMFTYMRFNAEFTF | 18 |
| CVA10 VP1-18 (127-145) | YMRFNAEFTFVTTTDNGEA | 19 |
| CVA10 VP1-19 (136-153) | FVTTTDNGEARPYILQYM | 18 |
| CVA10 VP1-20 (144-161) | EARPYILQYMYVPPGAPK | 18 |
| CVA10 VP1-21 (152-169) | YMYVPPGAPKPTGRDAFQ | 18 |
| CVA10 VP1-22 (160-178) | PKPTGRDAFQWQTATNPSV | 19 |
| CVA10 VP1-23 (169-186) | QWQTATNPSVFVKLTDPP | 18 |
| CVA10 VP1-24 (177-194) | SVFVKLTDPPAQVSVPFM | 18 |
| CVA10 VP1-25 (185-202) | PPAQVSVPFMSPASAYQW | 18 |
| CVA10 VP1-26 (193-210) | FMSPASAYQWFYDGYPTF | 18 |
| CVA10 VP1-27 (201-217) | QWFYDGYPTFGQHPETS | 17 |
| CVA10 VP1-28 (208-223) | PTFGQHPETSNTTYGL | 16 |
| CVA10 VP1-29 (214-229) | PETSNTTYGLCPNNMM | 16 |
| CVA10 VP1-30 (220-237) | TYGLCPNNMMGTFAVRVV | 18 |
| CVA10 VP1-31 (228-244) | MMGTFAVRVVSRKASQL | 17 |
| CVA10 VP1-32 (235-252) | RVVSRKASQLKLQTRVYM | 18 |
| CVA10 VP1-33 (243-260) | QLKLQTRVYMKLKHVRAW | 18 |
| CVA10 VP1-34 (251-266) | YMKLKHVRAWVPRPIR | 16 |
| CVA10 VP1-35 (257-274) | VRAWVPRPIRSQPYLLKN | 18 |
| CVA10 VP1-36 (265-279) | IRSQPYLLKNFPNYD | 15 |
| CVA10 VP1-37 (270-284) | YLLKNFPNYDSSKVT | 15 |
| CVA10 VP1-38 (275-292) | FPNYDSSKVTNSARDRSS | 18 |
| CVA10 VP1-39 (283-298) | VTNSARDRSSIKQANM | 18 |
| EV71 VP1-2 (9-26) | ESSIGDSVSRALTQALPA | 18 |
| EV71 VP2-19 (139-155) | TGTEDTHPPYKQTQPGA | 17 |
| EV71 VP3-8 (57-73) | NVPTNATSLMERLRFPV | 17 |

^a^ The number spectrum in the parentheses represents the amino acids spanning of each peptide.

**Figure S1**


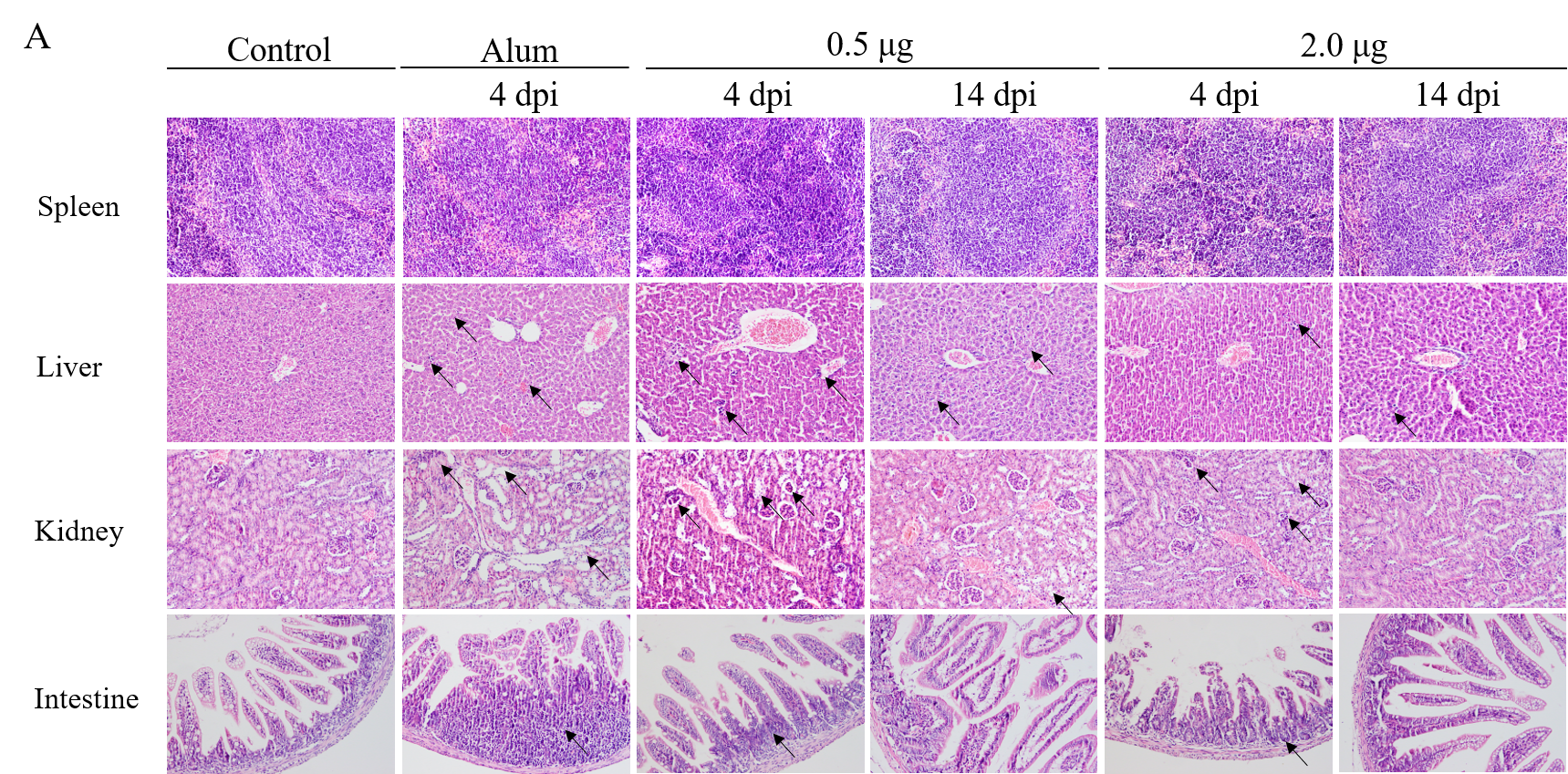


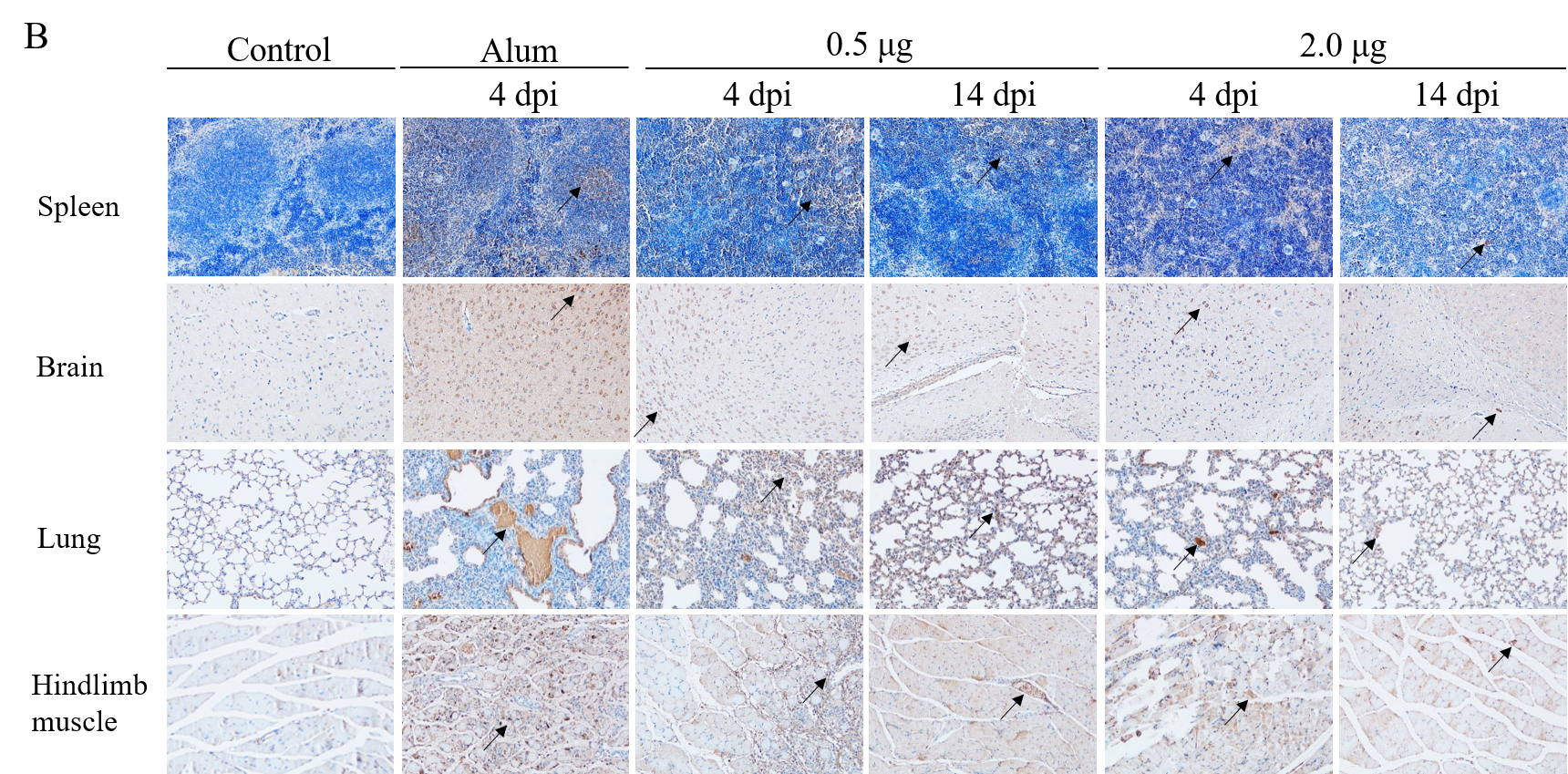


**Figure S1.** The analysis of the histopathological (H&E) and immunohistochemical (IHC) examination of tissues from immunized-challenged mice (200×). Immunized Kunming mice were inoculated i.p. with CV-A10-M14. The Alum groups were euthanized at 4 days post-challenge, and mice in the vaccinated groups were euthanized at 4 and 14 day-post-challenge. The healthy Kunming mice at the age of 28 days served as the negative control. For the convenience of figure presentation, “days post-challenge” was represented by “dpi”. (A) The spleen, liver, kidney and intestine were sectioned and observed. (B) IHC assays for the organs of spleen, brain, lung and hindlimb muscle were performed using a rabbit polyclonal antibody against CV-A10 whole virus. Black arrows indicated representative pathological damage (A) and expression of viral protein (B).

**Figure S2**


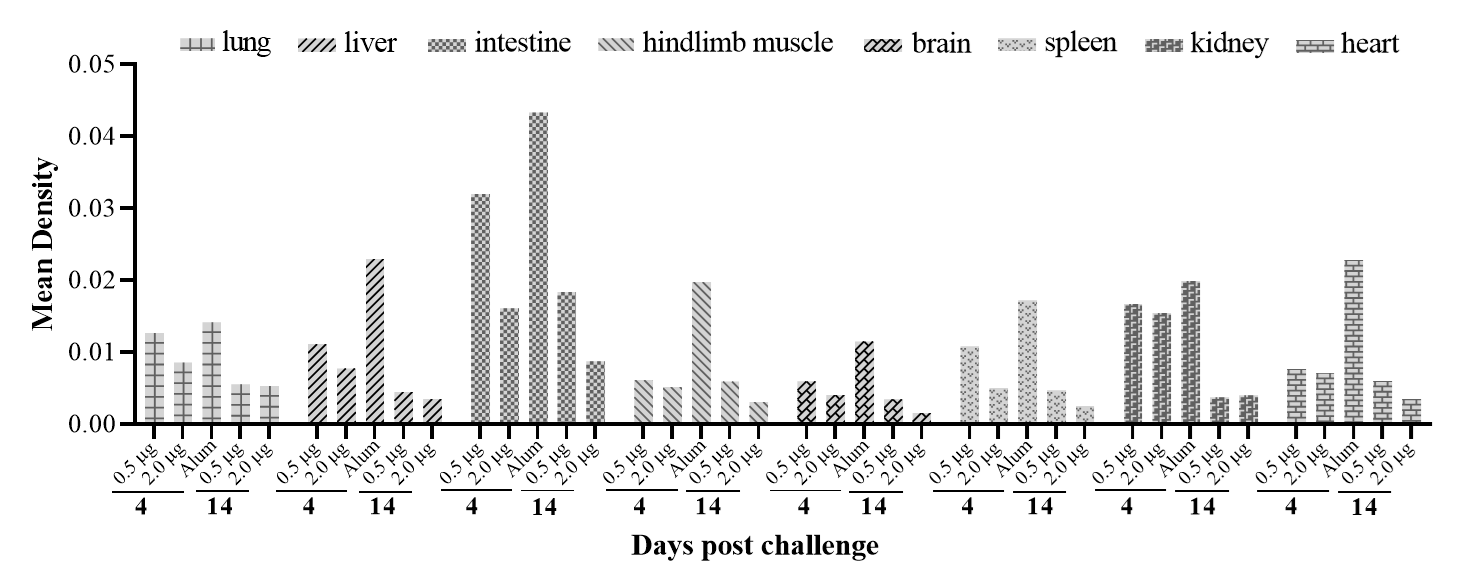


**Figure S2.** The quantitative analysis of the immunohistochemical (IHC) examination of organs from immunized-challenged mice. An IPP6.0 image analysis system was applied to calculate the mean optical density based on the positive area from IHC images in Figure 8B and supplemental Figure S1B.
